# Long-term Evaluation of Dentin Bonding Properties of the Photoinitiator System Contained in Universal Adhesives Used in Fiber-Post Luting Procedures

**DOI:** 10.3290/j.jad.b4786551

**Published:** 2023-12-20

**Authors:** Pedro Henrique de Aguiar Moreira, Patrick Pereira Garcia, Myrella do Nascimento Correia, Narla dos Reis Bacelar Chaves, Camilo Pulido, Michel Wendlinger Cantanhede Ferreira, Alessandra Reis, Fabiana Suelen Figuerêdo de Siqueira, Alessandro D. Loguercio, Andres Felipe Millan Cardenas

**Affiliations:** a MS Student, Postgraduate Program in Dentistry, CEUMA University, São Luis, Maranhão, Brazil. Performed bond strength experiments.; b MS Student, Postgraduate Program in Dentistry, CEUMA University, São Luis, Maranhão, Brazil. Co-wrote and proofread the manuscript.; c PhD Student, Postgraduate Program in Dentistry, CEUMA University, São Luis, Maranhão, Brazil. Co-wrote and proofread the manuscript.; d Professor, Department of Restorative Dentistry and Dental Materials, School of Dentistry, University of San Francisco de Quito USFQ, Quito, Ecuador. Performed nanoleakage experiments, co-wrote and proofread the manuscript.; e PhD Student, Department of Restorative Dentistry, State University of Ponta Grossa, Ponta Grossa, Paraná, Brazil. Performed nanoleakage experiments and co-wrote the manuscript.; f Professor of Department of Restorative Dentistry, State University of Ponta Grossa, Ponta Grossa, Paraná, Brazil. Provided consulting for statistical analysis and contributed substantially to the discussion.; g Professor, Postgraduate Program in Dentistry, CEUMA University, São Luis, Maranhão, Brazil. Research idea, designed testing assembly, contributed substantially to the discussion, co-wrote and proofread the manuscript.; h Professor, Department of Restorative Dentistry, State University of Ponta Grossa, Ponta Grossa, Paraná, Brazil. Provided consulting for statistical analysis, contributed substantially to the discussion, proofread the manuscript.; i Professor, Department of Postgraduate Program in Dentistry, CEUMA University, São Luis, Maranhão, Brazil. Research idea, designed testing assembly, contributed substantially to the discussion, co-wrote and proofread the manuscript.

**Keywords:** fiber-post cementation, resin cement, fiber post, nanoleakage.

## Abstract

**Purpose:**

This study evaluated the long-term push-out bond strength (PBS) and nanoleakage (NL) of universal adhesives with different photo-initiator systems in the root canals of teeth in which fiber posts were luted.

**Materials and Methods:**

One-hundred twenty endodontically treated human premolars were randomly divided into 12 groups based on the following factors: adhesives (Scotchbond Universal [SBU], Ambar Universal [AMB], and Ambar Universal APS [AMB-APS]); adhesive strategy (etch-and-rinse and self-etch), and time of testing (immediately vs after 2 years). The posts were cemented, sectioned into slices, tested for PBS at 0.5 mm/min, and examined for NL using scanning electron microscopy immediately thereafter or after 2 years of water storage. Data were evaluated using a four-way ANOVA (root thirds vs time vs universal adhesive vs adhesive strategies) and Tukey’s test (α = 5%).

**Results:**

For both evaluation times, AMB-APS demonstrated no significant difference in the PBS or NL when different radicular thirds were compared (p > 0.05). However, for SBU and AMB, the cervical third demonstrated higher PBS and lower NL than those of the apical third at both time points (p < 0.0001). AMB-APS exhibited higher PBS and lower NL in the apical third in comparison with SBU and AMB (p < 0.0001). SBU and AMB displayed a significant decrease in the PBS and increased NL after 2 years (p < 0.0001), whereas AMB-APS demonstrated no significant signs of degradation even after 2 years of water storage (p > 0.05).

**Conclusion:**

Independent of the root third evaluated, the universal adhesive containing APS photo-initiator system demonstrated bonding stability at the adhesive interface between the root canal and fiber posts, even after 2 years of water storage.

Glass-fiber posts are widely used in clinical situations involving extensive loss of coronal structure and endodontically treated roots owing to their favorable mechanical properties compared to metal cast posts.^2,17^ Root-canal dentin has a complex heterogenicity of dentinal tubule size and distribution, in addition to varying amounts of water, as a function of depth.^10^ Moreover, ensuring moisture control in the root canal is a very complicated procedure.^15^ Additionally, operative limitations such as visibility, especially in the apical third, may render clinicians more susceptible to making mistakes during the adhesive process.^24,37,40^

Moreover, light attenuation through fiber posts^23^ determines the effectiveness of polymerizing the resin luting agent and adhesive. The latter could be a determining factor, due to lower irradiance reaching the deepest portions of the root canal as a function of the distance to the light polymerization unit.^60^ Consequently, despite the presence of translucent posts, the quantity of light that reaches the apical third of the post space could be inadequate for achieving thorough curing of the cement in deeper root regions.^25,23^ This inadequacy results in compromised mechanical properties and bonding.^11,48^ Thus, the use of self-cure (SC) or dual-cure (DC) resin cement in conjunction with adhesives is commonly recommended for bonding light-transmitting or translucent fiber posts to the root canal wall.^11,25^

Consequently, DC resin cement has gained widespread popularity for fiber-post cementation by combining the benefits of both light curing, which enables rapid initial polymerization, and self-curing, which ensures chemical polymerization in areas with reduced accessibility. However, several studies have demonstrated that inadequately light-cured portions of DC or SC cements are incompatible with simplified and acidic adhesives. The incompatibility arises owing to the negative chemical interactions between the acidic resin monomers in simplified adhesives that could impair the polymerization of SC and DC, which is initiated by conventional peroxide-amine binary redox initiators.^61,62^ Even when the adhesive layer is light cured before applying the resin cement, the same unfavorable chemical reaction occurs with the partially polymerized adhesive layer and co-initiators used in SC and DC resin cement.^59^ Hence, unexpected debonding of restorations is a common observation.^39^

To address the unfavorable acid-base reaction mentioned above, certain adhesives have been enhanced by the inclusion of a second bottle containing an SC activator. The addition of SC activator serves two purposes: first, it reduces chemical incompatibility, and second, it ensures complete polymerization in the deeper regions of the root canal. Typically, the SC activators contain sodium sulfinate salts, which are believed to interact with air-inhibited monomers. The interaction results in the generation of phenyl or benzene sulfonyl free-radicals, which initiate the polymerization process via the SC mechanism of the adhesive bonding resin.^6,31^

Unfortunately, the incorporation of a chemical initiator into several commercially available adhesives has proven to be insufficient for achieving adequate curing. Some studies have demonstrated that adhesives cured in the SC mode either do not cure or exhibit a very low degree of conversion,^7,21^ which has a detrimental impact on the bond strengths, as highlighted by previous studies.^37,49^

Therefore, light curing of DC adhesives and resin cement is commonly recommended for improving the degree of conversion and enhancing the overall mechanical properties.^7,20^ Additionally, light curing of DC resin cement contributes to increased bond strengths when compared with only self-curing of DC resin cement.^8,20,36,37,49^

An alternative approach involves utilizing a light-curing adhesive that undergoes chemical curing through the action of a catalyst present in the resin cement (“touch-and-cure” mechanism). Chemical polymerization of the adhesive is initiated upon contact with a compatible dual-activated resin cement.^28^ Typically, the system incorporates an accelerator that facilitates rapid chemical polymerization by interacting with a specific chemical initiator present in the dual-activated resin cement.^29^ Although the mechanical properties and bond strength of light-activated adhesives to dentin are notably enhanced by touch-polymerization activators,^18,19^ they are further improved when accompanied by an extended light-irradiation duration.^19,36-38^

Considering the challenges in polymerization and moisture control in the root canal, mainly in the apical third, some manufacturers have added more reactive and hydrophilic photo-initiators to the composition of the adhesives. The most commonly used photo-initiation system is composed of a photosensitizer, such as camphorquinone (CQ), which may absorb light, and different types of amines and co-initiators that interact with the excited CQ and promote free-radical production with subsequent initiation of polymerization.^58^ However, CQ possesses hydrophobic properties, creating an antagonistic effect when combined with adhesive solutions containing hydrophilic components necessary for interaction with tooth substrates. This hampers adequate infiltration of the adhesive into the oversaturated dentin surface.^67^

Efforts to reduce moisture content during the luting process do not reduce the water content within the dentin matrix. The dentin surface can be such that the adhesive can be physically separated into hydrophobic and hydrophilic-rich phases.^57^ Hence, the water content of the dentin and the hydrophobicity of some photo-initiators (such as CQ) could be considered the main factors decreasing monomer conversion, owing to the poor interaction between them and the hydrophilic-rich matrix.^12^ Several studies have demonstrated that adhesives containing a hydrophilic photoinitiator yield superior outcomes in terms of polymerization efficacy and bond strength to dentin compared to CQ-containing adhesives.^16,34,40,43^

Recently, an in-vitro study^12^ demonstrated that the mechanism included in an advanced polymerization system (APS system) could increase the immediate degree of conversion and, consequently, the bonding performance of fiber posts luted into radicular dentin, especially in the apical third, when an adhesive containing an APS system was compared with the same adhesive without APS.^14^ According to the manufacturer, the APS photo-initiator system reduces the amount of CQ, which is balanced by the synergistic action of a combination of several photo- and co-initiators. This would contribute to the reduction in incompatibility in the hydrophilic-rich phase promoted by the hydrophobicity of CQ/amines. However, as degradation of the adhesive interface is a phenomenon that could increase over time, long-term evaluation of these is necessary.

Thus, the present study aimed to evaluate the use of three universal adhesives with different photo initiator systems, applied with etch-and-rinse (ER) and self-etch (SE) strategies on radicular dentin, and evaluate this interaction through fiber post luting (push-out bond strength [PBS] and nanoleakage) immediately and after 2 years of water storage. The null hypotheses tested were that the evaluated bonding characteristics of the interface between the post to radicular dentin would not be affected by the 1) root thirds, 2) time, 3) universal adhesives, and 4) adhesive strategies.

## MATERIALS AND METHODS

### Sample Preparation

The study was approved by the ethics committee of the State University of Ponta Grossa, PR, Brazil (protocol 2.408.873). One hundred twenty caries-free human mandibular premolars extracted within the past 6 months were selected and stored in distilled water at 4°C. The study sample included sound teeth without root cracks or severe root damage that had not undergone previous endodontic treatment. The teeth had a root length of 14 ± 1 mm, as measured from the cementoenamel junction. The tooth crowns were removed using a diamond saw (Isomet 1000, Buehler; Lake Bluff, IL, USA). A single calibrated operator, a specialist in endodontics and experienced in all the endodontic techniques used, performed the root canal preparation and obturation according to the endodontic technique described by Vilas-Boas et al.^65^ Root canals were prepared 1 mm shorter than the apical foramen with Reciproc R40 (VDW; München, Germany) and rinsed using 1% sodium hypochlorite (10 ml in total). Subsequently, the smear layer was removed using 5 ml of 17% ethylenediaminetetraacetic acid for 3 min and flushed with 10 ml of distilled water. The canals were dried using absorbent paper points (VDW), then filled with AH Plus (Dentsply Sirona; Konstanz, Germany) using the single-cone technique (R40, Reciproc). The cervical opening was sealed (glass-ionomer cement Maxxion R, FGM; Joinville, SC, Brazil) and the teeth were stored at 37°C in 100% humidity for 7 days.

### Fiber-Post Cementation

The filling material was removed from the coronal 10 mm of the canal (leaving 4 mm of gutta-percha in the apical third of the root) using a #3 Largo bur (Dentsply Sirona). The post space was prepared using a #2 bur (White Post DC #2, FGM) of 10 mm length, followed by irrigation (10 ml of distilled water) and drying with absorbent paper points. The specimens were randomly divided into 12 groups (n=10) according to one of three adhesives: Scotchbond Universal (SBU), 3M Oral Care (St Paul, MN, USA); Ambar Universal (AMB), FGM; and Ambar Universal APS (AMB-APS), FGM. Two adhesive strategies (ER and SE) were employed for each system, and the storage times were 24 h or after 2 years in distilled water. Product information and application modes are listed in Table 1. Before cementation, the glass-fiber posts were sectioned horizontally in the coronal region using a water-cooled diamond cutting instrument to reduce the post length to 13 mm. While 10 mm was cemented inside the root canal, the coronal 3 mm served as a guide to standardize the distance between the light-curing device and the cervical section of the root. All the posts were cleaned with gauze and immersed in 70% alcohol for 5 s the before luting procedure.

**Table 1:** Adhesive (manufacturer/batch number), composition, and application mode according to adhesive strategy

Adhesive, code (batch number)	Composition	Adhesive Strategies	
		Self-etch (SE)	Etch-and-rinse (ER)
Ambar Universal APS AMB-APS (070817)	10-MDP, methacrylic monomers, photo-initiator APS, CQ, silica nanoparticles, ethanol, co-initiators, and stabilizers	Apply two layers with a microbrush for 20 s (10 s per layer). Evaporate the adhesive solvent by using a gentle air stream for 10 s. Light cure for 10 s at 1200 mW/cm^2^.	Etch dentin with phosphoric acid for 15 s. Rinse with water spray to remove the acid. Evaporate the adhesive solvent, using a gentle air stream. Apply the adhesive in self-etching mode.
Ambar Universal AMB (200416)	10-MDP, hydrophilic methacrylic monomers, ethanol, silanized silicon dioxide, CQ, coinitiators and stabilizers	Apply two layers with a microbrush for 20 s (10 s each layer). Evaporate the adhesive solvent using a gentle air stream for 10 s. Light cure for 10 s at 1200 mW/cm^2^.	Etch dentin with phosphoric acid for 15 s. Rinse with water spray to remove the acid. Evaporate the adhesive solvent using a gentle air stream. Apply the adhesive in self-etching mode.
Scotchbond Universal SBU (1630600505)	10-MDP, HEMA, bis-GMA, decamethylene dimethacrylate, ethanol, silane-treated silica, water, copolymer of acrylic and itaconic acid, CQ, dimethylaminobenzoate(-4)	Apply the adhesive actively for 20 s and, if necessary, reapply the adhesive. Air dry for 5 s until the adhesive does not move and the solvent evaporates completely. Light cure for 10 s to 1200 mW/cm^2^.	Etch dentin with phosphoric acid for 15 s. Rinse with water spray to remove the acid. Evaporate the adhesive solvent using a gentle air stream. Apply the adhesive in self-etching mode.

All adhesives were applied according to manufacturers’ instructions. 10-MDP: 10-methacryloyloxydecyl dihydrogen phosphate; APS: advanced polymerization system; CQ: camphorquinone; HEMA: 2-hydroxyethyl methacrylate; bis-GMA: bisphenol A-glycidyl methacrylate.

After adhesive application, a dual-cure resin cement (All Cem, shade A2, FGM) was inserted using a Centrix syringe (DFL; Rio de Janeiro, RJ, Brazil). Double-tapered glass-fiber posts (White Post DC #2, FGM) with a smooth surface, a diameter of 1.8 mm at the top and 1.05 mm in the apical area, and a length of 20 mm, were inserted immediately and light polymerized for 40 s (1200 mW/cm^2^, Valo, Ultradent; South Jordan, UT, USA).

### Specimen Preparation and Measurement

After storage in water at 37ºC for 7 days, the specimens were sectioned perpendicular to their long axis under water cooling using a cutting machine (Isomet 1000, Buehler; Lake Bluff, IL, USA) at a speed of 300 rpm, to obtain two 1-mm-thick slices of each root third of the specimens, so that six disks were obtained from each root. The samples were then stored in distilled water at 37°C; after 24 h, three disks from each root (cervical, middle, and apical) and from each experimental group were subjected to PBS and nanoleakage tests. The other three disks from each root (cervical, medium, and apical) were stored in distilled water at 37°C for 2 years. The aqueous storage medium containing 0.5% chloramine-T was changed weekly. For all slices, both sides were photographed at 40X magnification to measure the coronal and apical diameters of the posts in order to calculate individual bonding areas (Image J, National Institutes of Health; Bethesda, MD, USA).

### Push-out Bond Strength Test (PBS)

After 24 h or 2 years of water storage, the PBS test was performed to measuring the bond strength of the fiber post to root canal dentin. The cervical side of each test specimen was placed in contact with a special device (Odeme; Joaçaba, SC, Brazil) coupled to the base of a universal testing machine (Instron 3342; Canton, MA, USA). Loading was performed at a crosshead speed of 0.5 mm/min until the post was completely dislodged from the root slice. A metal tip was used to apply a compressive force until post debonding. The diameter of the metallic tips was compatible with the diameter of the post in each third (1.6, 1.2, and 0.8 mm diameter in the cervical, medium, and apical third, respectively), being slightly smaller to allow the compressive force only in contact with the post surface.^55^ The maximum value obtained in kilogram-force was used to calculate the bond strength in MPa using the following formula: BS = π(R + r)[(h^2^ + (R – r)^2^] 0.5, where π = 3.14, R = coronal post radius, r = apical post radius, and h = root section thickness in mm. The debonded specimens were observed under 40X magnification using a stereomicroscope loupe (SZ61, Olympus America; Center Valley, PA, USA) to categorize the failure mode into three types: 1) adhesive at the post-cement interface or at the cement/dentin interface; 2) adhesive mixed at the post/cement/dentin interface; and 3) cohesive in the dentin, cement, or post.

### Nanoleakage Test

One bonded slice per tooth from each storage time that was not used in the PBS test was selected for examination. The slices were immersed in a 50 wt% ammoniacal silver nitrate solution for 48 h and photodeveloped for 8 h under indirect fluorescent light. After polishing with wet 600-, 1000-, 1200-, 1500-, 2000-, 2500-, and 4000-grit silicon carbide paper, each slab was ultrasonically cleaned, mounted, and sputter-coated in a vacuum evaporator (SCD 050, Balzers Union; Balzers, Liechtenstein). The entire surface was examined using a scanning electron microscope (VEGA 3 TESCAM, Shimadzu; Tokyo, Japan). First, the slices were examined at a magnification of 600X to identify the central region, then several micrographs were obtained at a magnification of 1000X. The percentage of nanoleakage at the bonded interface was measured using ImageJ software.

### Statistical Analysis

The PBS and nanoleakage data for all slices from the same tooth were averaged for statistical analysis. After evaluating data normality using the Kolmogorov-Smirnov test and the equality of variances using the Bartlett test, the data were evaluated by four-way ANOVA (root thirds vs time vs universal adhesives vs adhesive strategies) and Tukey’s test (α = 5%). All analyses were performed using SPSS (Statistical Package for the Social Science) version 17.0 (SPSS; Chicago, IL, USA).

## RESULTS

### Push-out Bond Strengths

In all the experimental groups, 99% of the specimens demonstrated adhesive (at the cement/dentin interface) and adhesive-mixed failures (Table 2). Only 1% of the specimens failed cohesively (Table 2 ). The PBSs are presented in Table 3. Only a three-way ANOVA cross-product interaction among root thirds, time, and universal adhesives revealed a statistically significant effect in terms of PBS (p < 0.0001), as did the main factors of root third (p < 0.0001), adhesive (p < 0.0001), and storage time (p < 0.0001). No significant differences were observed between the adhesive strategies for any of the tested adhesives (p = 0.33).

**Table 2:** Fracture mode (%) of all experimental groups

Experimental groups			Cervical			Medium			Apical		
			A	M	C	A	M	C	A	M	C
Ambar Universal APS	SE	Immediate	28 (93)	2 (7)	0 (0)	29 (97)	1 (3)	0 (0)	30 (100)	0 (0)	0 (0)
		2 years	30 (100)	0 (0)	0 (0)	30 (100)	0 (0)	0 (0)	30 (100)	0 (0)	0 (0)
	ER	Immediate	29 (97)	1 (3)	0 (0)	27 (90)	1 (3)	2 (7)	30 (100)	0 (0)	0 (0)
		2 years	30 (100)	0 (0)	0 (0)	30 (100)	0 (0)	0 (0)	30 (100)	0 (0)	0 (0)
Ambar Universal	SE	Immediate	30 (100)	0 (0)	0 (0)	30 (100)	0 (0)	0 (0)	29 (97)	1 (3)	0 (0)
		2 years	30 (100)	0 (0)	0 (0)	30 (100)	0 (0)	0 (0)	30 (0)	0 (0)	0 (0)
	ER	Immediate	29 (97)	1 (3)	0 (0)	28 (93)	2 (7)	0 (0)	30 (100)	0 (0)	0 (0)
		2 years	30 (100)	0 (0)	0 (0)	30 (100)	0 (0)	(0)	29 (97)	1 (3)	0 (0)
Single Bond Universal	SE	Immediate	29 (97)	1 (3)	0 (0)	30 (100)	0 (0)	0 (0)	29 (97)	1 (3)	0 (0)
		2 years	30 (100)	0 (0)	0 (0)	30 (100)	0 (0)	0 (0)	30 (100)	0 (0)	0 (0)
	ER	Immediate	27 (90)	3 (10)	0 (0)	27 (90)	3 (10)	0 (0)	29 (97)	1 (3)	0 (0)
		2 years	30 (100)	0 (0)	0 (0)	30 (100	0 (0)	0 (0)	29 (97)	1 (3)	0 (0)

A: adhesive failure; M: mixed failure; C: cohesive failure.

**Table 3:** Means ± SD of push-out bond strengths (MPa) obtained in all experimental groups

Experimental groups			Cervical	Medium	Apical
Ambar Universal APS	SE	Immediate	13.1 ± 2.3^a,b^	12.7 ± 2.6^a,b^	13.2 ±2.2^a^
		2 years	11.8 ± 2.3^a,b^	11.4 ± 2.8^a,b^	11.7 ±2.6^a,b^
	ER	Immediate	13.8 ± 2.7^a^	12.9 ± 2.7^a,b^	13.4 ±2.4^a^
		2 years	11.9 ± 2.7^a,b^	10.9 ± 2.4^b,c^	11.8 ±2.3^a,b^
Ambar Universal	SE	Immediate	14.5 ± 2.7^a^	10.8 ± 2.7^b^	9.2 ±3.5^b,c^
		2 years	10.2 ± 1.8^b,c^	7.7 ± 2.1^d^	6.2 ±1.9^d^
	ER	Immediate	14.6 ± 2.7^a^	11.6 ± 2.7^a,b^	9.2 ±2.4^b,c^
		2 years	10.4 ± 2.8^b,c^	8.6 ± 2.0^c,d^	7.5 ±2.0^d^
Single Bond Universal	SE	Immediate	13.3 ± 2.7^a^	10.8 ± 2.6^b^	8.1 ±2.3^c^
		2 years	8.5 ± 2.6^c^	6.8 ± 2.1^d^	4.6 ±2.0^d^
	ER	Immediate	14.1 ± 2.7^a^	10.9 ± 2.8^b^	9.3 ±3.5^b,c^
		2 years	8.8 ± 2.2^c^	6.2 ± 2.5^d^	5.3 ±2.4^d^

Similar superscript letters indicate no significant difference among the groups (four-way ANOVA and Tukey’s test; p = 0.05).

Immediately (24 h) and after 2 years of water storage, AMB-APS exhibited no significant difference in PBS comparing different radicular thirds (Table 3, p > 0.05). In contrast, for SBU and AMB, the cervical third demonstrated higher push-out bond strengths than did the apical third (Table 3, p < 0.0001). Furthermore, at both 24 h and 2 years, AMB-APS exhibited significantly higher PBS values in the apical third in comparison with the corresponding values observed for SBU and AMB (Table 3, p < 0.0001).

When the two evaluation times were compared, no significant decrease in PBS was observed in any third of AMB-APS samples (Table 3, p > 0.05). However, a significant decrease in PBS was observed after 2 years of water storage, in comparison with the immediate values, and for all the thirds of SBU and AMB specimens (Table 3, p < 0.0001).

### Nanoleakage Evaluation

**Fig 1:**
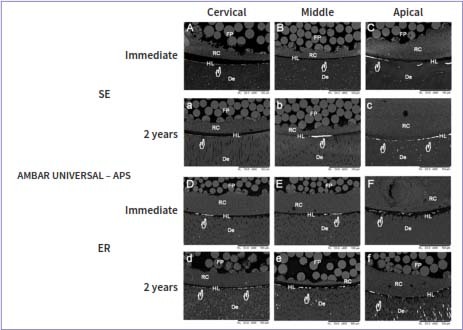
Representative back-scattered SEM micrographs (600X) of the fiber-post/radicular-dentin interface for Ambar universal APS examined for nanoleakage immediately and after 2 years of water storage. Although silver nitrate deposits (white hands) are observed in all the root thirds, reduced silver nitrate uptake is apparent in the immediately tested specimens (white hands). When the immediate results are compared to 2 years, only a slight increase is observed after 2 years of water storage. SE: self-etch; ER: etch-and-rinse; FP: fiber post; RC: resin cement; HL: hybrid layer; De: radicular dentin.

**Fig 2:**
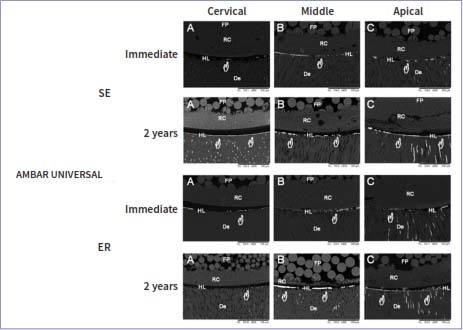
Representative back-scattered SEM micrographs (600X) of the fiber-post/ radicular-dentin interface for Ambar universal tested immediately and after 2 years of water storage. Independent of the time of storage, higher silver nitrate uptake (white hands) can be observed in the apical third, with more evident signs of degradation at the base of the hybrid and adhesive layers (water trees) after 2 years of water storage. SE: self-etch; ER: etch-and-rinse; FP: fiber post; RC: resin cement; HL: hybrid layer; De: radicular dentin.

**Fig 3:**
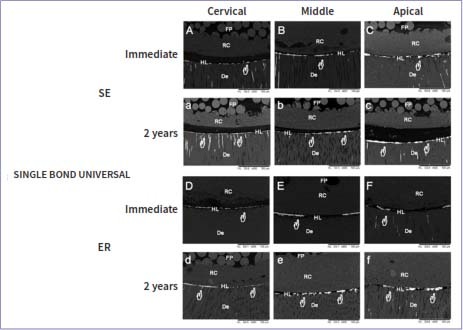
Representative back-scattered SEM micrographs (600X) of the fiber-post/radicular-dentin interface of Single Bond Universal tested immediately and after 2 years of water storage. Silver traces (white hands) are observed in all the root thirds, especially in the apical thirds. However, this is more evident after 2 years of water storage. Usually, immediately-tested restorations exhibit less silver nitrate uptake than after 2 years of water storage. P: fiber post; RC: resin cement; HL: hybrid layer; De: radicular dentin.

The nanoleakage values are presented in Table 4, and scanning electron micrographs of each experimental group are displayed in Figs 1–3. For the nanoleakage values, only a three-way ANOVA cross-product interaction among root thirds vs time vs universal adhesives revealed a statistically significant effect (p < 0.001), as did the main factors root third (p < 0.001), adhesive (p < 0.001), and storage time (p < 0.001). There were no significant differences between the adhesive strategies for any of the tested adhesives (p = 0.75).

**Table 4:** Means ± SD of nanoleakage values (%) obtained in all experimental groups

Experimental groups			Cervical	Medium	Apical
Ambar Universal APS	SE	Immediate	7.5 ± 1.8^a^	7.7 ± 1.7^a^	7.1 ± 2.1^a^
		2 years	8.7 ± 2.0^ a^	8.4 ± 1.8^ a^	9.5 ± 1.1^a,b^
	ER	Immediate	7.7 ± 1.7^ a^	8.1 ± 1.9^a^	8.0 ± 1.7^a^
		2 years	7.8 ± 1.2^ a^	8.0 ± 1.7^a^	9.3 ± 1.5^a,b^
Ambar Universal	SE	Immediate	6.8 ± 2.1^ a^	8.1 ± 3.0^a^	10.9 ± 2.7^b^
		2 years	11.0 ± 1.7^b^	12.7 ± 2.2^ b,c^	14.4 ± 1.5^c,d^
	ER	Immediate	7.3 ± 2.3^a^	9.6 ± 1.7^a,b^	11.3 ± 1.3^b^
		2 years	11.5 ± 1.1^b^	13.2 ± 1.5^c^	14.1 ± 1.4^c,d^
Single Bond Universal	SE	Immediate	7.3 ± 1.3^a^	11.2 ± 2.2^b^	12.4 ± 1.3^b,c^
		2 years	11.7 ± 2.0^ab^	13.4 ± 1.6^c^	16.7 ± 1.6^d^
	ER	Immediate	7.9 ± 1.9^a^	10.2 ± 0.9^b^	11.8 ± 0.7^c^
		2 years	12.4 ± 2.2^b,c^	14.1 ± 1.1^c,d^	16.3 ± 1.7^d^

Similar superscript letters indicate no significant difference among the groups (four-way ANOVA and Tukey’s test; p = 0.05).

Immediately and after 2 years of water storage, AMB-APS showed no significant difference in nanoleakage values when different radicular thirds were compared (Table 4, p > 0.05). In contrast, for SBU and AMB, the cervical third showed less nanoleakage than did the apical third (Table 4, p < 0.001). In addition, AMB-APS showed significantly less nanoleakage in the apical third than did apical thirds of SBU and AMB (Table 4, p < 0.001).

Comparing the two evaluation times, no significant decrease in nanoleakage was observed in any third when AMB-APS was evaluated (Table 4, p > 0.05). However, a significant increase in nanoleakage was observed for all the thirds and both adhesives (SBU and AMB) after 2 years of water storage compared to the immediate values (Table 4, p < 0.001).

## DISCUSSION

The results of the present study demonstrated that for SBU and AMB, the cervical third exhibited higher PBS and lower silver nitrate deposits than the apical third, both immediately and after 2 years of storage, leading to the rejection of the first and second null hypotheses. The adhesive performance in different radicular dentin regions is not uniform in some cases, owing to anatomical differences according to the root canal length. For instance, the cervical third has higher PBS partly because this portion of the root has the most organized and coronal-like dentin tubules.^47^ In addition, its proximity to the curing light may result in higher radiant exposure and better mechanical behavior.^14,60,69^ Although translucent fiber posts were used, light transmission was attenuated by the post,^25,23^ so that polymerization of the adhesive may have been compromised in the apical third, as observed in several studies.^11,12,15,60^

Moreover, the degree of conversion can be impaired by operative limitations during the luting process, mainly because clinicians are unable to ensure moisture control in the dentin, especially in the apical third.^11^ In the presence of water, methacrylate adhesives may undergo phase separation into hydrophobic and hydrophilic phases^57^ during photopolymerization. Adhesive phase separation inhibits the formation of a resistant bond interface.^67^ Thus, the behavior of photo-initiators in the presence of water is critical for the success of fiber-post luting to root dentin. As described previously by several authors,^16,14,57,67^ a slightly more hydrophobic photo-initiator contained in some adhesives could impair its interaction with the hydrophilic-rich polymer matrix, compromising monomer conversion.^45^

In addition, these areas of suboptimal conversion within the polymer matrix showed significantly increased nanoleakage (silver nitrate uptake), thus jeopardizing the PBS of SBU and AMB and leading to the rejection of the third null hypothesis. When AMB and SBU were compared, significant differences were observed, which could be related to the presence of high-molecular-weight monomers, such as bisphenol A-glycidyl methacrylate and polyalkenoic acid copolymer in SBU. For instance, it is well known that polyalkenoic acid copolymers do not dissolve well in adhesive solutions. Hence, a separate phase producing many globules within the polymer of the adhesive layer^16,63^ could have a detrimental effect on the bonding properties of root dentin.

In contrast, AMB-APS exhibited no significant differences in PBS and nanoleakage when different radicular thirds were compared. AMB-APS contains an alternative photo-initiator system, called APS. Unfortunately, the exact composition of each universal adhesive is proprietary information. However, according to the manufacturer, in this system, the reduction in the amount of CQ is balanced by the combination of several photo- and co-initiators that, when combined, could complement each other. Therefore, several mechanisms should be involved in a system such as APS. Some authors have demonstrated that the addition of a third or more hydrophilic component to the hydrophobic photo-initiator system is an effective alternative because, taking into consideration that active radicals are produced by both hydrophobic and hydrophilic initiators, these different phases can improving polymerization of both hydrophilic and hydrophobic domains.^45,68^ Also, the addition of different hydrophilic co-initiators to a photo-initiator system increases the photoreactivity, owing to the ability to maintain a more stable energy level during the excited state of CQ.^68^ Moreover, this system can stabilize the energy level during the excitation of CQ, allowing its recycling during the process and potentializing its action.^27^ All the processes acting simultaneously could reduce incompatibility in the hydrophilic-rich phase promoted by the hydrophobicity of CQ/amines and consequently increase the degree of conversion at the root/dentin interface.^12^ Lower silver nitrate uptake and higher PBS of AMB-APS, mainly in the apical third of the root in comparison with SBU and AMB, confirmed that AMB-APS enhanced polymerization even in a more hydrophilic environment.

Another possible explanation for the lack of significant differences in PBS and nanoleakage between different radicular thirds is that AMB-APS was used together with the resin cement from the same manufacturer; it is plausible that the polymerization of AMB-APS involved a “touch-and-cure” mechanism.^28,29^ Thus, the adhesive should have contained an accelerator that promoted rapid chemical polymerization with a specific chemical initiator when in contact with the AllCem resin cement.

According to Kim et al,^30^ touch-and-cure activators can generally be categorized as two types based on their key components: aryl sulfinic acid sodium salt-based activators and aryl borate salt-based activators. Unfortunately, as the exact composition of each adhesive/resin cement is proprietary information, it was not possible to confirm this hypothesis.

It is worth mentioning that the manufacturer of SBU adhesive claims that its performance is enhanced when it comes in contact with the resin cement RelyX Ultimate (3M Oral Care). According to the manufacturer, the resin cement incorporates an integrated chemically curing activator for SBU.^1^ Unfortunately, in the present study, RelyX Ultimate resin cement was not used in conjunction with the SBU, which could have negatively influenced the performance of SBU. However, the mechanism has not been previously confirmed, as several studies that evaluated the adhesive performance of SBU in association with RelyX Ultimate demonstrated poor bond strength when SBU and RelyX Ultimate were not light cured.^5,36-38^ For example, Luhrs et al,^36^ evaluated the association of SBU + RelyX Ultimate in different curing modes with the bonding of CAD/CAM composite restorations to dentin. Those authors demonstrated that when SBU + RelyX Ultimate was light cured, values as high as 31.7 MPa were observed. However, when only one part of the systems was light cured or both underwent self-curing (SC) only, values ranged from 1.4 to 6.2 were observed. This indicated that the association of SBU with RelyX Ultimate was highly dependent on light curing, and therefore did not contribute to the improvement of the adhesive properties of the root canal, especially in the apical third, as observed in the present study.

Regarding the results after 2 years of water storage, some degradation of the adhesive interface between the post and root canal is expected to occur, mainly when using universal adhesives.^11^ The new generation of adhesives comprises one-step simplified products: the hydrophobic and hydrophilic components are mixed with an organic solvent without a separate hydrophobic layer.^42,64^ In highly hydrophilic adhesives, complete solvent elimination does not occur,^12^ and the presence of residual volatile solvent directly influences the degree of conversion of the hybrid and adhesive layers.^50^ Therefore, incomplete polymerization of methacrylate materials occurs, increasing the presence of residual monomers, which may have a plasticizing effect on the polymer, thereby altering the physical and mechanical properties of the adhesive.^26^ Moreover, the presence of unreacted monomer can render the polymer matrix more susceptible to hydrolytic degradation, compromising its longevity.^68^

Indeed, the results of the present study demonstrated a decrease in PBS and an increase in the nanoleakage for SBU and AMB when immediate data were compared with data observed after 2-year water storage. Interestingly, no differences were observed for the AMB-APS when the evaluation times were compared. As previously mentioned, the polymerization ability can reduce degradation over time and improve the physical and mechanical properties of a system. In combination with a more hydrophilic composition, the APS system could maintain more stable energy levels during the excited state of CQ, enhancing its action and increasing the photoreactivity of the hydrophilic monomers.^27^ This explained their performance and stability over time.^33^

Although no consensus exists in the literature on the most suitable mode of application (ER or SE strategy) when using universal adhesives in dentin root^3,9,54^ or coronal dentin,^22,56^ the results of previous studies demonstrated that the performance of these adhesives did not depend on the adhesive strategy used,^3,9,54^ which is consistent with the results of the present study; thus, the fourth null hypothesis was accepted. This is a clinically relevant result, since clinicians can choose one universal adhesive and use it according to his/her preference or clinical demand.^41,42^

Despite the long-term clinical success achieved with the use of intraradicular fiber posts for the rehabilitation of endodontically treated teeth, several have revealed certain disadvantages. One of them is the additional removal of sound tissue needed for fitting the post into the root canal,^32^ thereby affecting the overall biomechanical behavior of the finally restored tooth.^51^ Therefore, postless restorations have been suggested.^13,52^ Future clinical studies should be conducted to compare the use of intraradicular fiber posts with no posts in endodontically treated teeth.

The present study had several limitations. First, long-term storage was employed using only water. Nevertheless, the storage of adhesive specimens in distilled water is a well-accepted method for evaluating the degradation of the bonding interfaces,^35,53^ mainly because the presence of water is crucial for their deterioration, but this storage method does not simulate all clinical situations.^4,44,46,66^ Second, as the resin cement recommended by the manufacturer (3M Oral Care) was not used with SBU, the complete potential bonding for SBU could not be fully elucidated here. Third, since the correlation of results of in-vitro studies with the clinical performance of several bonding materials is imperfect, it was not possible to extrapolate the results of the present study to clinical practice. Therefore, despite the superior bonding properties observed in the present study for AMB-APS used to lute fiber posts when compared with SBU and AMB, mainly after 2 years of water storage, clinical studies are needed to confirm these findings. Fourth, only one dual-curing resin cement was used in this study. Owing to differences in the mechanical properties between various such cements, the present experimental design should be repeated with a different dual-cure resin cement.

## CONCLUSION

After 2 years of water storage and independent of the root third evaluated, the universal adhesive containing an advanced photo-initiator system for polymerization demonstrated bonding stability at the fiber-post/root-canal interface.

## Clinical relevance:

The introduction of an advanced polymerization system in universal adhesives could promote the adhesive stability of fiber posts in root canals.
